# Supplementary material for: German recommendations for physical activity and physical activity promotion in adults with noncommunicable diseases
Source: Int J Behav Nutr Phys Act. 2020 Feb 5;17:12. doi: 10.1186/s12966-020-0919-x (PMC7003325; doi:10.1186/s12966-020-0919-x)
Supplement: Supplementary file 1 — Additional file 1. Criteria for Quality Assessment and Content Analysis of Physical Activity Recommendations. This file lists the 28 criteria used to assess methodological quality and analyse motion-related content. [file 12966_2020_919_MOESM1_ESM.pdf]

Additional file 1. Criteria for Quality Assessment and Content Analysis of Physical Activity Recommendations.

Domain A: Scope and purpose

1. The target group (age groups, patient groups, etc.) for which the recommendation is to apply is clearly described.
2. The recommendations indicate target group prerequisites that must be full-filled prior to the a) start of a physical exercise program or b) an increase in physical activity (Keyword: pre-exercise evaluation).
3. The user target group of the recommendation is defined.

Domain B1: Methodological quality of the development

4. Systematic methods were used in the search for evidence (or existing guidelines).
5. The criteria for selecting evidence are clearly described.
6. The recommendations take into account the current state of research.
7. The methods used to formulate the recommendations are clearly described.
8. The link between recommendations and the underlying evidence is explicit.

Domain B2§: Methodological quality of the development

9. Systematic methods have been used in the search for existing guidelines.

10 The selection of recommendations used as evidence sources (so-called source recommendations) is transparent and explicitly justified.

11. The quality of the source recommendations was reviewed.
12. Systematic research was carried out to update the source recommendations.
13. Modifications of source recommendations are clearly identified and justified.

Domain C: Content Clarity and Differentiation

14. The recommendations are specific and unambiguous.
15. Recommendations factored health benefits such as activity, participation & individual contextual factors in.
16. Potential health benefits through (recommendation) compliance are described precisely (if given: outome, size, & evidence level).
17. Side effects and risks were considered while designing the recommendations
18. Recommendations on activity dosage include exact details on intensity, duration, frequency.
19. Recommendations differentiate between health-enhancing physical activity & exercise programs/ training, basic activities
20. Recommendations include details on physical activity dose-response rates.
21. Recommendations include a precise description of the implementation of motor abilities (endurance, strength, coordination etc.) .
22. The recommendations contain a description of the usage of motor abilities or the type of bodily movement (e.g. Running, Nordic-Walking or Gymnastics).
23. Recommendations consider physical activity in a variety of domains, such as sport(s), leisure, recuperation, household & gardening.
24. Recommendations indicate how to reduce sedentary time OR regularly break up longer lasting sedentary periods.
25. Recommendations refer to individual prerequisites (e.g. fitness-level, physical baseline, mental prerequisites).

Domain D: Layout

26. Key recommendations are easily identified within the recommendations.
27. The most current evidence/state-of-art is summarized is briefly (within recommendations).
28. Recommendation transfer to the target group is supported by existing instruments/ materials, such as: illustrations, associated online-portals.

Note: § Questions 9-14 contain criteria which only apply if existing recommendations or guidelines - so-called source recommendations - have been used in the development of the recommendations to be evaluated.
