# Supplementary material for: German recommendations for physical activity and physical activity promotion in adults with noncommunicable diseases
Source: Int J Behav Nutr Phys Act. 2020 Feb 5;17:12. doi: 10.1186/s12966-020-0919-x (PMC7003325; doi:10.1186/s12966-020-0919-x)
Supplement: Supplementary file 2 — Additional file 2. Quality checklist for the systematic reviews of reviews regarding the effectiveness/efficacy of PA promoting interventions. This file lists the criteria used to evaluate the papers reporting PA promotion interventions with regard to their effectiveness/efficacy. [file 12966_2020_919_MOESM2_ESM.pdf]

Additional file 2. Quality checklist for the systematic reviews of reviews regarding the effectiveness/efficacy of PA promoting interventions.

**Criterium**	**Explanation**	**Meta-Analysis**	**Review**
Search terms and links of the search terms are stated explicitly (1).	Without this information the study is not transparent.	Yes	Yes
It is stated which databases were searched (1).	Without this information the study is not transparent.	Yes	Yes
There is a diagram for included/excluded studies (1).	This is considered as standard.	Yes	Yes
The included studies are shown in tables (min. design, measures, outcomes) (1).	This is considered as standard.	Yes	Yes
There are estimations of the size/effects of different bias factors (1).	This is considered as standard.	Yes	Yes
The problem of dependent measurements in the aggregation is discussed or dealt with (by exclusion or by statistical treatment of the dependencies) (2, 3).	Dependent measurements in studies lead to an overestimation of effects. As a minimum, this problem should be addressed. Methods for aggregating dependent measurements are on the market but are not used often.	Yes	When appro-priate
Effect sizes and not only ordinal assessments of primary study results are reported (3).	For meta-analyses inacceptable. However, for many reviews an averaged effect size can be reported (but not always).	Yes	When appro-priate
Furthermore, (only or mainly) effect sizes without a statistical bias are reported (Hedges'g or log-OR) (3).	Changes in percentage values show – depending on the baseline value – a bias. Because of that, summaries of unbiased mean values are preferable. For reviews, this depends on the data availability.	Yes	When appro-priate
More than 5 primary studies per analysis are reported (except in subgroup-analyses, see below) (3).	For 5 or less studies a summary depends strongly on the single studie. These reviews/meta-analysis are less useful.	Yes	Yes
An analysis of the publication bias was conducted (e.g. funnel plot or variance analyses) (3, 4).	This is a standard fort he estimation of the publication bias in meta-analyses.	Yes	No
Forest plots are reported (3).	This is a standard in meta-analyses. In reviews forest plots should be reported when appropriate data are available.	Yes	When appro-priate
A check of the study heterogeneity was conducted (l-square and p-value) (3).	This is a standard in meta-analyses. In reviews this should be discussed at least regarding the existence of heterogeneity.	Yes	When appro-priate
Heterogeneous results are not only reported, but also discussed (3).	This is a standard in meta-analyses. In reviews this should be discussed at least regarding the existence of heterogeneity.	Condi-tionally (l12=1)	When appro-priate
For clarifying heterogeneous results, meta-regressions or subgroup-analyses are conducted (3).	This is a standard in meta-analyses. Not applicable for reviews.	Condi-tionally (l12=1)	When appro-priate
It is evident that the results are/were not only caused by one/a few big study/-ies (3).	This should be checked both in meta-analyses and in reviews.	Yes	Yes
Very small but significant effects (|g| < 0,10; |LOR| < 0,10) are discussed regarding their relevance (1).	Such results are possible in meta-analyses. It is a problem that both model violations and dominant studies result in such effects. A discussion is essential. Only reporting „significances" is not helpful.	Yes	No
Insignificant or very small but heterogeneous effects are analysed by using sensitivity analyses, meta-regressions or subgroup-analyses (1).	This should be done in meta-analyses.	Yes	No

References

1. Higgins JP, Green S. Cochrane Handbook for Systematic Reviews of Interventions. Version 5.1.0 [updated March 2011].
2. Hedges LV, Tipton E, Johnson MC. Robust variance estimation in meta-regression with dependent effect size estimates. Res Synth Methids. 2010;1(1):39-65.
3. Borenstein M, Hedges LV, Higgins JPT, Rothstein HR. Introduction to Meta-Analysis. 2009. Wile. doi:10.1002/9780470743386
4. Sterne JA, Egger M, Smith GD. Systematic reviews in health care: investigating and dealing with publication and other biases in meta-analysis. BMJ. 2001;323(7304):101-5.
