# Supplementary material for: German recommendations for physical activity and physical activity promotion in adults with noncommunicable diseases
Source: Int J Behav Nutr Phys Act. 2020 Feb 5;17:12. doi: 10.1186/s12966-020-0919-x (PMC7003325; doi:10.1186/s12966-020-0919-x)
Supplement: Supplementary file 3 — Additional file 3. Quality ratings of the physical activity recommendations and additional ressources (meta-analysis and reviews on physical activity recommendations) that were additional included without explicit quality ratings for seven noncommunicable diseases. This file lists the retrieved articles and their quality ratings leading to the decision on which articles the German Recommendations for Physical Activity for Adults with NCD were developed. [file 12966_2020_919_MOESM3_ESM.pdf]

Additional file 3.Quality ratings of the physical activity recommendations and additional ressources (meta-analysis and reviews on physical activity recommendations) that were additional included without explicit quality ratings for seven noncommunicable diseases.

Author(s)	Domain A: Scope and purpose	Domain B1: Methodological accuracy for recommendations	Domain B2: Methodological accuracy for reviews of recommend-dations	Domain C: Clarity and differentiation of content	Domain D: Structure	Inclusion
**Osteoarthrosis**						
Beckwee et al. [1]	75	70	50	56	50	Yes
Bennell et al. [2]	75	45	nr	65	50	No
Fernandes et al. [3]	92	85	nr	71	75	Yes
Hochberg et al. [4]	83	95	nr	54	67	Yes
Larmer et al. [5]	75	95	75	54	67	Yes
Lu et al. [6]	67	95	nr	56	75	Yes
McAlindon et al. [7]	83	100	nr	56	67	Yes
Nelson et al. [8]	75	75	80	50	50	Yes
Stoffer et al. [9]	83	75	65	38	83	Yes
Thomas [10]	50	35	nr	46	42	No
Uthmann et al. [11]	75	100	nr	58	67	Yes
Additional ressources						
Escalante et al. [12]						Yes
Fransen et al. [13]						Yes
Fransen et al. [14]						Yes
Kelley et al. [15]						Yes
Zacharias et al. [16]						Yes
Zhang et al. [17]						Yes
**Type 2-Diabetes Mellitus**						
Colberg et al. [18]	83	55	nr	77	83	No
Franz et al. [19]	58	40	nr	35	58	No
Geidl et al. [20]	100	80	nr	83	100	Yes
Misra et al. [21]	83	50	nr	48	83	No
O,Hagan et al. [22]	58	55	70	44	58	Yes
Ryden et al. [23]	67	75	nr	46	75	Yes
Sigal et al. [24]	100	95	nr	67	67	Yes
**COPD**						
Abdool-Gaffar et al. [25]	92	40	25	50	33	No
Gupta et al. [26]	83	65	65	46	50	Yes
Iepsen et al. [27]	92	90	65	40	25	Yes
Jones et al. [28]	83	30	nr	38	33	No
Marciniuk et al. [29]	92	90	100	54	75	Yes
Pan et al. [30]	92	70	nr	27	25	Yes
Additional ressources						
Ng et al. [31]						Yes
Garvey et al. [32]						Yes
**Clincial stalbe coronary heart disease**						
Fihn et al. [33]	92	85	25	71	75	Yes
Misra et al. [21]	75	65	55	35	50	Yes

Perez-Terzic [34]	58	40	nr	67	50	No
Additional ressources						
Oldridge et al. [35]						Yes
Seron et al. [36]						Yes
**Clinical depression**						
Berk et al. [37]	67	60	nr	44	67	Yes
Danielsson et al. [38]	67	80	nr	58	50	Yes
Nystrom et al. [39]	83	80	nr	54	50	Yes
Park et al. [40]	67	60	nr	35	50	Yes
Perraton et al. [41]	83	75	nr	54	50	Yes
Ranjbar et al. [42]	92	75	nr	58	67	Yes
Additional ressources						
Knapen et al. [43]						Yes
**Strokel**						
Billinger et al. [44]	83	60	nr	92	42	Yes
Borschmann [45]	42	70	55	46	42	No
Bryer et al. [46]	67	60	nr	33	50	Yes
Gallanagh et al. [47]	67	65	35	46	33	Yes
Klinke et al. [48]	58	85	nr	46	58	No
McDonnell et al. [49]	58	25	25	27	33	No
Pang et al. [50]	67	75	nr	71	67	Yes
Pollock et al. [51],	67	70	nr	27	50	Yes
Poltawski et al. [52]	50	70	60	25	25	No
Steib et al. [53]	67	65	80	58	42	Yes
Zehr et al. [54]	92	70	nr	48	67	Yes
Additional ressources						
Adamson et al [55]						Yes
Coupar et al. [56]						Yes
Coupar et al. [57]						Yes
Lubetzky-Vilnai et al. [58]						Yes
Saltychev et al. [59]						Yes
**Chronic unspecific low back pain**						
Chilibeck et al. [60]	75	70	nr	65	83	Yes
Haladay et al. [61]	67	65	nr	42	58	Yes
Kristensen et al. [62]	50	60	nr	25	50	No
Ribaud et al. [63]	75	60	nr	60	67	Yes
Pillastrini et al. [64]	67	85	70	40	58	Yes
Steele et al. [65]	67	75	nr	48	50	Yes
Additional ressources						
Dagenais et al. [66]						Yes
Fersum et al. [67]						Yes
Ladeira [68]						Yes
Standaert et al. [69]						Yes
Van Middelkoop et al. [70]						Yes
Wang et al. [71]						Yes

Abbreviation. nr: not relevant.

References (Additional file 3)

1. Beckwee D, Vaes P, Cnudde M, Swinnen E, Bautmans I. Osteoarthritis of the knee: why does exercise work? A qualitative study of the literature. Ageing Res Rev. 2013;12:226–36. doi:10.1016/j.arr.2012.09.005.
2. Bennell KL, Dobson F, Hinman RS. Exercise in osteoarthritis: moving from prescription to adherence. Best Pract Res Clin Rheumatol. 2014;28:93–117. doi:10.1016/j.berh.2014.01.009.
3. Fernandes L, Hagen KB, Bijlsma, Johannes W J, Andreassen O, Christensen P, Conaghan PG, et al. EULAR recommendations for the non-pharmacological core management of hip and knee osteoarthritis. Ann Rheum Dis. 2013;72:1125–35. doi:10.1136/annrheumdis-2012-202745.
4. Hochberg MC, Altman RD, April KT, Benkhalti M, Guyatt G, McGowan J, et al. American College of Rheumatology 2012 recommendations for the use of nonpharmacologic and pharmacologic therapies in osteoarthritis of the hand, hip, and knee. Arthritis Care Res (Hoboken). 2012;64:465–74.
5. Larmer PJ, Reay ND, Aubert ER, Kersten P. Systematic review of guidelines for the physical management of osteoarthritis. Arch Phys Med Rehabil. 2014;95:375–89. doi:10.1016/j.apmr.2013.10.011.
6. Lu M, Su Y, Zhang Y, Zhang Z, Wang W, He Z, et al. Effectiveness of aquatic exercise for treatment of knee osteoarthritis: Systematic review and meta-analysis. Z Rheumatol 2015. doi:10.1007/s00393-014-1559-9.
7. McAlindon TE, Bannuru RR, Sullivan MC, Arden NK, Berenbaum F, Bierma-Zeinstra SM, et al. OARSI guidelines for the non-surgical management of knee osteoarthritis. Osteoarthritis Cartilage. 2014;22:363–88. doi:10.1016/j.joca.2014.01.003.
8. Nelson AE, Allen KD, Golightly YM, Goode AP, Jordan JM. A systematic review of recommendations and guidelines for the management of osteoarthritis: the chronic osteoarthritis management initiative of the U.S. bone and joint initiative. Semin Arthritis Rheum. 2014;43:701–12. doi:10.1016/j.semarthrit.2013.11.012.
9. Stoffer MA, Smolen JS, Woolf A, Ambrozic A, Berghea F, Boonen A, et al. Development of patient-centred standards of care for osteoarthritis in Europe: the eumusc.net-project. Ann Rheum Dis 2014. doi:10.1136/annrheumdis-2014-206176.
10. Thomas JL. Helpful or harmful? Potential effects of exercise on selected inflammatory conditions. Phys Sportsmed. 2013;41:93–100. doi:10.3810/psm.2013.11.2040.
11. Uthman OA, van der Windt, D. A., Jordan JL, Dziedzic KS, Healey EL, Peat GM, Foster NE. Exercise for lower limb osteoarthritis: systematic review incorporating trial sequential analysis and network meta-analysis. BMJ. 2013;347:f5555. doi:10.1136/bmj.f5555.
12. Escalante Y, Garcia-Hermoso A, Saavedra JM. Effects of exercise on functional aerobic capacity in lower limb osteoarthritis: a systematic review. J Sci Med Sport. 2011;14:190–8. doi:10.1016/j.jsams.2010.10.004.
13. Fransen M, McConnell S, Harmer AR, Van der Esch, Martin, Simic M, Bennell KL. Exercise for osteoarthritis of the knee. Cochrane Database Syst Rev. 2015;1:CD004376. doi:10.1002/14651858.CD004376.pub3.
14. Fransen M, McConnell S, Hernandez-Molina G, Reichenbach S. Exercise for osteoarthritis of the hip. Cochrane Database Syst Rev. 2014;4:CD007912. doi:10.1002/14651858.CD007912.pub2.
15. Kelley GA, Kelley KS, Hootman JM. Effects of exercise on depression in adults with arthritis: a systematic review with meta-analysis of randomized controlled trials. Arthritis Res Ther. 2015;17:21. doi:10.1186/s13075-015-0533-5.
16. Zacharias A, Green RA, Semciw AI, Kingsley, M I C, Pizzari T. Efficacy of rehabilitation programs for improving muscle strength in people with hip or knee osteoarthritis: a systematic review with meta-analysis. Osteoarthritis Cartilage. 2014;22:1752–73. doi:10.1016/j.joca.2014.07.005.
17. Zhang W, Nuki G, Moskowitz RW, Abramson S, Altman RD, Arden NK, et al. OARSI recommendations for the management of hip and knee osteoarthritis: part III: Changes in evidence following systematic cumulative update of research published through January 2009. Osteoarthritis Cartilage. 2010;18:476–99. doi:10.1016/j.joca.2010.01.013.

18. Colberg SR, Sigal RJ, Fernhall B, Regensteiner JG, Blissmer BJ, Rubin RR, et al. Exercise and type 2 diabetes: The American College of Sports Medicine and the American Diabetes Association: Joint position statement: Diabetes Care. 2010;33:e147-e167. doi:10.2337/dc10-9990.
19. Franz M.J., Powers M.A., Leontos C., Holzmeister L.A., Kulkarni K., Monk A., et al. The evidence for medical nutrition therapy for type 1 and type 2 diabetes in adults. J Am Diet Assoc. 2010;110:1852–89.
20. Geidl W, Pfeifer K. Körperliche Aktivität und körperliches Training in der Rehabilitation des Typ-2-Diabetes. Rehabilitation. 2011;50:255–65. doi:10.1055/s-0031-1280805.
21. Misra A, Nigam P, Hills AP, Chadha DS, Sharma V, Deepak KK, et al. Consensus physical activity guidelines for Asian Indians. Diabetes Technol Ther. 2012;14:83–98. doi:10.1089/dia.2011.0111.
22. O'Hagan C, Vito G de, Boreham, Colin A. G. Exercise Prescription in the Treatment of Type 2 Diabetes Mellitus. Sports Med. 2013;43:39–49. doi:10.1007/s40279-012-0004-y.
23. Rydén L, Grant PJ, Anker SD, Berne C, Cosentino F, Danchin N, et al. ESC guidelines on diabetes, pre-diabetes, and cardiovascular diseases developed in collaboration with the EASD - summary. Diab Vasc Dis Res. 2014;11:133–73. doi:10.1177/1479164114525548.
24. Sigal RJ, Armstrong MJ, Colby P, Kenny GP, Plotnikoff RC, Reichert SM, Riddell MC. Physical activity and diabetes. Canad J Diabet. 2013;37:S40–S44. doi:10.1016/j.jcjd.2013.01.018.
25. Abdool-Gaffar MS, Ambaram A, Ainslie GM, Bolliger CT, Feldman C, Geffen L, et al. Guideline for the management of chronic obstructive pulmonary disease--2011 update. S Afr Med J. 2011;101:63–73.
26. Gupta D, Agarwal R, Aggarwal AN, Maturu VN, Dhooria S, Prasad KT, et al. Guidelines for diagnosis and management of chronic obstructive pulmonary disease: Joint ICS/NCCP (I) recommendations. Lung India. 2013;30:228–67. doi:10.4103/0970-2113.116248.
27. Iepsen UW, Jorgensen KJ, Ringbaek T, Hansen H, Skrubbeltrang C, Lange P. A Systematic Review of Resistance Training Versus Endurance Training in COPD. J Cardiopulm Rehabil Prev. 2015;35:163–72. doi:10.1097/HCR.0000000000000105.
28. Jones R, Gruffydd-Jones K, Pinnock H, Peffers S-J, Lawrence J, Scullion J, et al. Summary of the consultation on a strategy for services for chronic obstructive pulmonary disease (COPD) in England. Prim Care Respir J. 2010;19 Suppl 2:S1-S17. doi:10.4104/pcrj.2010.00081.
29. Marciniuk DD, Brooks D, Butcher S, Debigare R, Dechman G, Ford G, et al. Optimizing pulmonary rehabilitation in chronic obstructive pulmonary disease--practical issues: a Canadian Thoracic Society Clinical Practice Guideline. Can Respir J. 2010;17:159–68.
30. Pan L, Guo YZ, Yan JH, Zhang WX, Sun J, Li BW. Does upper extremity exercise improve dyspnea in patients with COPD? A meta-analysis. Respir Med. 2012;106:1517–25. doi:10.1016/j.rmed.2012.08.002.
31. Ng, Bobby H P, Tsang, Hector W H, Ng, Bacon F L, So C-t. Traditional Chinese exercises for pulmonary rehabilitation: evidence from a systematic review. J Cardiopulm Rehabil Prev. 2014;34:367–77. doi:10.1097/HCR.0000000000000062.
32. Garvey C, Fullwood MD, Rigler J. Pulmonary rehabilitation exercise prescription in chronic obstructive lung disease: US survey and review of guidelines and clinical practices. J Cardiopulm Rehabil Prev. 2013;33:314–22. doi:10.1097/HCR.0b013e318297fea4.
33. Fihn SD, Gardin JM, Abrams J, Berra K, Blankenship JC, Dallas AP, et al. 2012 ACCF/AHA/ACP/AATS/PCNA/SCAI/STS Guideline for the diagnosis and management of patients with stable ischemic heart disease: a report of the American College of Cardiology Foundation/American Heart Association Task Force on Practice Guidelines, and the American College of Physicians, American Association for Thoracic Surgery, Preventive Cardiovascular Nurses Association, Society for Cardiovascular Angiography and Interventions, and Society of Thoracic Surgeons. J Am Coll Cardiol. 2012;60:e44-e164. doi:10.1016/j.jacc.2012.07.013.
34. Perez-Terzic CM. Exercise in cardiovascular diseases. PM R. 2012;4:867–73. doi:10.1016/j.pmrj.2012.10.003.
35. Oldridge N. Exercise-based cardiac rehabilitation in patients with coronary heart disease: meta-analysis outcomes revisited. Future Cardiol. 2012;8:729–51. doi:10.2217/fca.12.34.
36. Serón P, Lanas F, Ríos E, Bonfill X, Alonso-Coello P. Evaluation of the quality of clinical guidelines for cardiac rehabilitation: a critical review. J Cardiopulm Rehabil Prev. 2015;35:1–12. doi:10.1097/HCR.0000000000000075.
37. Berk M, Sarris J, Coulson CE, Jacka FN. Lifestyle management of unipolar depression. Acta Psychiatr Scand Suppl. 2013:38–54. doi:10.1111/acps.12124.

38. Danielsson L, Noras AM, Waern M, Carlsson J. Exercise in the treatment of major depression: a systematic review grading the quality of evidence. Physiother Theory Pract. 2013;29:573–85. doi:10.3109/09593985.2013.774452.
39. Nyström MBT, Neely G, Hassmen P, Carlbring P. Treating Major Depression with Physical Activity: A Systematic Overview with Recommendations. Cogn Behav Ther. 2015;44:341–52. doi:10.1080/16506073.2015.1015440.
40. Park S-C, Oh HS, Oh D-H, Jung SA, Na K-S, Lee H-Y, et al. Evidence-based, non-pharmacological treatment guideline for depression in Korea. J Korean Med Sci. 2014;29:12–22. doi:10.3346/jkms.2014.29.1.12.
41. Perraton LG, Kumar S, Machotka Z. Exercise parameters in the treatment of clinical depression: a systematic review of randomized controlled trials. J Eval Clin PRact. 2010;16:597–604. doi:10.1111/j.1365-2753.2009.01188.x.
42. Ranjbar E, Memari AH, Hafizi S, Shayestehfar M, Mirfazeli FS, Eshghi MA. Depression and Exercise: A Clinical Review and Management Guideline. Asian J Sports Med. 2015;6:e24055. doi:10.5812/asjsm.6(2)2015.24055.
43. Knapen J, Vancampfort D, Morien Y, Marchal Y. Exercise therapy improves both mental and physical health in patients with major depression. Disabil Rehabil. 2015;37:1490–5. doi:10.3109/09638288.2014.972579.
44. Billinger SA, Arena R, Bernhardt J, Eng JJ, Franklin BA, Johnson CM, et al. Physical activity and exercise recommendations for stroke survivors: a statement for healthcare professionals from the American Heart Association/American Stroke Association. Stroke. 2014;45:2532–53. doi:10.1161/STR.0000000000000022.
45. Borschmann K. Exercise protects bone after stroke, or does it? A narrative review of the evidence 2012. doi:10.1155/2012/103697.
46. Bryer A, Connor M, Haug P, Cheyip B, Staub H, Tipping B, et al. South African guideline for management of ischaemic stroke and transient ischaemic attack 2010: a guideline from the South African Stroke Society (SASS) and the SASS Writing Committee. S Afr Med J. 2010;100:747–78.
47. Gallanagh S, Quinn TJ, Alexander J, Walters MR. Physical activity in the prevention and treatment of stroke. ISRN Neurol. 2011;2011:953818. doi:10.5402/2011/953818.
48. Klinke ME, Hafsteinsdóttir TB, Hjaltason H, Jónsdóttir H. Ward-based interventions for patients with hemispatial neglect in stroke rehabilitation: a systematic literature review. Int J Nurs Stud. 2015;52:1375–403. doi:10.1016/j.ijnurstu.2015.04.004.
49. McDonnell MN. Physical activity following stroke. Arch Phys Med Rehabil. 2010;91:665–6. doi:10.1016/j.apmr.2009.12.007.
50. Pang, Marco Y C, Charlesworth SA, Lau, Ricky W K, Chung, Raymond C K. Using aerobic exercise to improve health outcomes and quality of life in stroke: evidence-based exercise prescription recommendations. Cerebrovasc Dis. 2013;35:7–22. doi:10.1159/000346075.
51. Pollock A, Farmer SE, Brady MC, Langhorne P, Mead GE, Mehrholz J, van Wijck F. Interventions for improving upper limb function after stroke. Cochrane Database Syst Rev. 2014;11:CD010820. doi:10.1002/14651858.CD010820.pub2.
52. Poltawski L, Abraham C, Forster A, Goodwin VA, Kilbride C, Taylor RS, Dean S. Synthesising practice guidelines for the development of community-based exercise programmes after stroke. Implement Sci. 2013;8:115. doi:10.1186/1748-5908-8-115.
53. Steib S, Schupp W. Therapiestrategien in der Schlaganfallnachsorge. Inhalte und Effekte. Nervenarzt. 2012;83:467–75. doi:10.1007/s00115-011-3396-2.
54. Zehr EP. Evidence-based risk assessment and recommendations for physical activity clearance: stroke and spinal cord injury. Appl Physiol Nutr Metab. 2011;36 Suppl 1:31. doi:10.1139/h11-055.
55. Adamson BC, Ensari I, Motl RW. Effect of Exercise on Depressive Symptoms in Adults With Neurologic Disorders: A Systematic Review and Meta-Analysis. Arch Phys Med Rehabil 2015. doi:10.1016/j.apmr.2015.01.005.
56. Coupar F, Pollock A, van Wijck F, Morris J, Langhorne P. Simultaneous bilateral training for improving arm function after stroke. Cochrane Database Syst Rev. 2010:CD006432. doi:10.1002/14651858.CD006432.pub2.
57. Coupar F, Pollock A, Legg LA, Sackley C, van Vliet P. Home-based therapy programmes for upper limb functional recovery following stroke. Cochrane Database Syst Rev. 2012;5:CD006755. doi:10.1002/14651858.CD006755.pub2.

58. Lubetzky-Vilnai A, Kartin D. The effect of balance training on balance performance in individuals poststroke: a systematic review. J Neurol Phys Ther. 2010;34:127–37. doi:10.1097/NPT.0b013e3181ef764d.
59. Saltychev M, SjogrenT T, Barlund E, Laimi K, Paltamaa J. Do aerobic exercises really improve aerobic capacity of stroke survivors? A systematic review and meta-analysis. Eur J Phys Rehabil Med. 2015.
60. Chilibeck PD, Vatanparast H, Cornish SM, Abeysekara S, Charlesworth S. Evidence-based risk assessment and recommendations for physical activity: arthritis, osteoporosis, and low back pain. Appl Physiol Nutr Metab. 2011;36 Suppl 1:79. doi:10.1139/h11-037.
61. Haladay DE, Miller SJ, Challis J, Denegar CR. Quality of systematic reviews on specific spinal stabilization exercise for chronic low back pain. J Orthop Sports Phys Ther. 2013;43:242–50. doi:10.2519/jospt.2013.4346.
62. Kristensen J, Franklyn-Miller A. Resistance training in musculoskeletal rehabilitation: a systematic review. Br J Sports Med. 2012;46:719–26. doi:10.1136/bjsm.2010.079376.
63. Ribaud A, Tavares I, Viollet E, Julia M, Herisson C, Dupeyron A. Which physical activities and sports can be recommended to chronic low back pain patients after rehabilitation? Ann Phys Rehabil Med. 2013;56:576–94. doi:10.1016/j.rehab.2013.08.007.
64. Pillastrini P, Gardenghi I, Bonetti F, Capra F, Guccione A, Mugnai R, Violante FS. An updated overview of clinical guidelines for chronic low back pain management in primary care. Joint Bone Spine. 2012;79:176–85. doi:10.1016/j.jbspin.2011.03.019.
65. Steele J, Bruce-Low S, Smith D. A review of the clinical value of isolated lumbar extension resistance training for chronic low back pain. PM R. 2015;7:169–87. doi:10.1016/j.pmrj.2014.10.009.
66. Dagenais S, Tricco AC, Haldeman S. Synthesis of recommendations for the assessment and management of low back pain from recent clinical practice guidelines. Spine J. 2010;10:514–29. doi:10.1016/j.spinee.2010.03.032.
67. Fersum KV, Dankaerts W, O'Sullivan PB, Maes J, Skouen JS, Bjordal JM, Kvale A. Integration of subclassification strategies in randomised controlled clinical trials evaluating manual therapy treatment and exercise therapy for non-specific chronic low back pain: a systematic review. Br J Sports Med. 2010;44:1054–62. doi:10.1136/bjsm.2009.063289.
68. Ladeira CE. Evidence based practice guidelines for management of low back pain: physical therapy implications. Rev Bras Fisioter. 2011;15:190–9.
69. Standaert CJ, Friedly J, Erwin MW, Lee MJ, Rechtine G, Henrikson NB, Norvell DC. Comparative effectiveness of exercise, acupuncture, and spinal manipulation for low back pain. Spine (Phila Pa 1976). 2011;36:30. doi:10.1097/BRS.0b013e31822ef878.
70. van Middelkoop M, Rubinstein SM, Verhagen AP, Ostelo RW, Koes BW, van Tulder, Maurits W. Exercise therapy for chronic nonspecific low-back pain. Best Pract Res Clin Rheumatol. 2010;24:193–204. doi:10.1016/j.berh.2010.01.002.
71. Wang X-Q, Zheng J-J, Yu Z-W, Bi X, Lou S-J, Liu J, et al. A meta-analysis of core stability exercise versus general exercise for chronic low back pain. PLoS One. 2012;7:e52082. doi:10.1371/journal.pone.0052082.
