# Supplementary material for: German recommendations for physical activity and physical activity promotion in adults with noncommunicable diseases
Source: Int J Behav Nutr Phys Act. 2020 Feb 5;17:12. doi: 10.1186/s12966-020-0919-x (PMC7003325; doi:10.1186/s12966-020-0919-x)
Supplement: Supplementary file 4 — Additional file 4. Quality rating of the reviews for developing the German Recommendations for PA Promotion. This table contains the quality rating of the reviews used to develop the German Recommendations for PA promotion. [file 12966_2020_919_MOESM4_ESM.pdf]

Additional file 4: Quality rating of the reviews for developing the German Recommendations for PA Promotion.

Review	Level of quality (QC)	Level of quality (AGREE)	Quality rating
Beinart et al 2013	66%	78%	Good
Bossen et al 2014	72%	73%	Good
Conn et al 2008	71%	69%	Medium
Cox et al 2013	75%	95%	Good
Cramp et al 2013	56%	76%	Medium
Iversen et al 2012	65%	70%	Medium
Leidy et al 2014	50%	56%	Low
Mansi et al 2014	70%	73%	Good
Mastellos et al 2014	80%	82%	Good
McGrane et al 2015	70%	73%	Good
Morris et al 2014	72%	78%	Good
Munro et al 2013	72%	77%	Good
O'Halloran et al 2014	73%	95%	Good
Orrow et al 2012	81%	86%	Good
Pavey et al 2011	56%	82%	Medium
Sargent et al 2012	53%	56%	Medium
Short et al 2013	66%	81%	Good
Ter Hoeve et al 2015	72%	78%	Good

References (Supplementary file 4)

Beinart, N. A.; Goodchild, C. E.; Weinman, J. A.; Ayis, S.; Godfrey, E. L. (2013): Individual and intervention-related factors associated with adherence to home exercise in chronic low back pain. A systematic review. In: Spine J. 13, S. 1940–1950. DOI: 10.1016/j.spinee.2013.08.027.

Bossen, D.; Veenhof, C.; Dekker, J.; Bakker, D. de (2014): The effectiveness of self-guided web-based physical activity interventions among patients with a chronic disease. A systematic review. In: Journal of physical activity & health 11, S. 665–677. DOI: 10.1123/jpah.2012-0152.

Conn, V. S.; Hafdahl, A. R.; Cooper, P. S.; Brown, L. M.; Lusk, S. L. (2009): Meta-analysis of workplace physical activity interventions. In: American journal of preventive medicine 37, S. 330–339. DOI: 10.1016/j.amepre.2009.06.008.

Cox, N. S.; Alison, J. A.; Holland, A. E. (2013): Interventions for promoting physical activity in people with cystic fibrosis. In: The Cochrane database of systematic reviews 12.

Cramp, F.; Berry, J.; Gardiner, M.; Smith, F.; Stephens, D. (2013): Health behaviour change interventions for the promotion of physical activity in rheumatoid arthritis. A systematic review. In: Musculoskeletal care 11, S. 238–247. DOI: 10.1002/msc.1049.

Iversen, M. D.; Brawerman, M.; Iversen, C. N. (2012): Recommendations and the state of the evidence for physical activity interventions for adults with rheumatoid arthritis. 2007 to present. In: Int. J. Clin. Rheumatol. 7, S. 489–503. DOI: 10.2217/ijr.12.53.

Leidy, N. K.; Kimel, M.; Ajagbe, L.; Kim, K.; Hamilton, A.; Becker, K. (2014): Designing trials of behavioral interventions to increase physical activity in patients with COPD. Insights from the chronic disease literature. In: Respiratory Medicine 108, S. 472–481. DOI: 10.1016/j.rmed.2013.11.011.

Mansi, S.; Milosavljevic, S.; Baxter, G. D.; Tumilty, S.; Hendrick, P. (2014): A systematic review of studies using pedometers as an intervention for musculoskeletal diseases. In: BMC Musculoskeletal Disorders 15. DOI: 10.1186/1471-2474-15-231.

Mastellos, N.; Gunn, L. H.; Felix, L. M.; Car, J.; Majeed, A. (2014): Transtheoretical model stages of change for dietary and physical exercise modification in weight loss management for overweight and obese adults. In: The Cochrane database of systematic reviews 2.

McGrane, N.; Galvin, R.; Cusack, T.; Stokes, E. (2015): Addition of motivational interventions to exercise and traditional Physiotherapy: a review and meta-analysis. In: Physiotherapy 101 (1), S. 1–12. DOI: 10.1016/j.physio.2014.04.009.

Morris, J. H.; Macgillivray, S.; McFarlane, S. (2014): Interventions to promote long-term participation in physical activity after stroke. A systematic review of the literature. In: Archives of Physical Medicine and Rehabilitation 95, S. 956–967. DOI: 10.1016/j.apmr.2013.12.016.

Munro, J.; Angus, N.; Leslie, S. J. (2013): Patient focused internet-based approaches to cardiovascular rehabilitation - a systematic review. In: Journal of Telemedicine and Telecare 19, S. 347–353. DOI: 10.1177/1357633X13501763.

O'Halloran, P. D.; Blackstock, F.; Shields, N.; Holland, A.; Iles, R.; Kingsley, M. et al. (2014): Motivational interviewing to increase physical activity in people with chronic health conditions. A systematic review and meta-analysis. In: Clinical Rehabilitation 28, S. 1159–1171. DOI: 10.1177/0269215514536210.

Orrow, G.; Kinmonth, A. L.; Sanderson, S.; Sutton, S. (2012): Effectiveness of physical activity promotion based in primary care. Systematic review and meta-analysis of randomised controlled trials. In: BMJ (Clinical research ed.) 344, S. 16. DOI: 10.1136/bmj.e1389.

Pavey, T. G.; Anokye, N.; Taylor, A. H.; Trueman, P.; Moxham, T.; Fox, K. R. et al. (2011): The clinical effectiveness and cost-effectiveness of exercise referral schemes. A systematic review and economic evaluation. In: Health technology assessment (Winchester, England) 15. DOI: 10.3310/hta15440.

Sargent, G. M.; Forrest, L. E.; Parker, R. M. (2012): Nurse delivered lifestyle interventions in primary health care to treat chronic disease risk factors associated with obesity. A systematic review. In: Obes. Rev. 13, S. 1148–1171. DOI: 10.1111/j.1467-789X.2012.01029.x.

Short, C. E.; James, E. L.; Stacey, F.; Plotnikoff, R. C. (2013): A qualitative synthesis of trials promoting physical activity behaviour change among post-treatment breast cancer survivors. In: Journal of Cancer Survivorship 7, S. 570–581. DOI: 10.1007/s11764-013-0296-4.

ter Hoeve, Nienke; Huisstede, Bionka M. A.; Stan, Henk J.; van Domburg, Ron T.; Sunamura, Madoka; van den Berg-Emons, Rita J. G. (2015): Does cardiac rehabilitation after an acute cardiac syndrome lead to changes in physical activity habits? Systematic review. In: PHYS. THER. 95, S. 167–179.
