# Supplementary material for: German recommendations for physical activity and physical activity promotion in adults with noncommunicable diseases
Source: Int J Behav Nutr Phys Act. 2020 Feb 5;17:12. doi: 10.1186/s12966-020-0919-x (PMC7003325; doi:10.1186/s12966-020-0919-x)
Supplement: Supplementary file 5 — Additional file 5. Quality criteria for the conceptualization, implementation and evaluation of PA promoting interventions. This table lists the general quality criteria for PA promotion interventions. [file 12966_2020_919_MOESM5_ESM.pdf]

Additional file 5. Quality criteria for the conceptualization, implementation and evaluation of PA promoting interventions.

**A) Quality of conceptualization**	
(1)	**Theoretical foundation**<br>- Scientific theories of health-related physical activity research<br>- Conceptual framework of health promotion (principles, fields of action)
(2)	**Multidimensionality**<br>- Behavioral and environmental components<br>- Interaction of the components (integrative approach)
(3)	**Relation to context**<br>- Individual and social factors<br>- Environmental and policy factors
(4)	**Relation to target group**<br>- Identification of the target group and its special characteristics<br>- Specification of needs and strengths of the target group
(5)	**Involvement of different stakeholders**<br>- Participation of the target group<br>- Participation of relevant professional groups and decision-makers
(6)	**Specification of goals and target behavior**
(7)	**Differentiated planning of the content-related and organizational process**<br>- Definition and justification of the content and methods of the intervention<br>- Specification of the project plan and temporal structure<br>- Specification of intervention components, frequency, intensity and duration<br>- Detailed description of the approach (manuals, protocols)
**B) Quality of implementation**	
(8)	**Involvement of different stakeholders**<br>- Target group(s) of the intervention<br>- Supporter / "social catalysts" (e.g. family)<br>- Professional groups (e.g. providers in the field of preventive medicine, decision maker)
(9)	**Empowerment of stakeholders / capacity development of organizations**<br>- Qualification/training<br>- Professional support
(10)	**Resources**<br>- Finances and time<br>- Infrastructures<br>- Quantity and quality of the involved staff<br>- Organizational/administrative commitment
(11)	**Networking/cooperation/partnership**<br>- Collaborative coordination of involved stakeholders/organizations<br>- Intersectoral alliances
(12)	**Consistence and adjustment**<br>- "Program loyalty"<br>- Compatibility<br>- Feasibility<br>- Adjustment to the implementation context
(13)	**Communication**<br>- Effective mechanisms for internal and external communication<br>- Transparency and openness
(14)	**Sustainability**<br>- Structural entrenchment<br>- Permanent takeover by involved stakeholders/community<br>- Continuation as an organizational routine
**C) Quality of evaluation**	
(15)	**Documentation and process evaluation**
(16)	**Evaluation of target group reach**<br>- Inclusion regarding age, gender, health status<br>- Cultural and social inclusion
(17)	**Outcome evaluation**<br>- Valid, reliable and sensitive methods/instruments<br>- Specified regarding strength, sustainability and generalizability of the effects<br>- Specified regarding health relevance<br>- Specified regarding negative effects
(18)	**Determine the cost/benefit relation**<br>- Costs in relation to behavior change<br>- Costs in relation to health benefits
